# The use of body weight support on ground level: an alternative strategy for gait training of individuals with stroke

**DOI:** 10.1186/1743-0003-6-43

**Published:** 2009-12-01

**Authors:** Catarina O Sousa, José A Barela, Christiane L Prado-Medeiros, Tania F Salvini, Ana MF Barela

**Affiliations:** 1 Department of Physical Therapy, Federal University of São Carlos, Rodovia Washington Luis, Km 235, CP, 676, 13656-905 São Carlos, SP, Brazil; 2 Graduate Program in Human Movement Sciences, Institute of Physical Activity and Sport Sciences, Cruzeiro do Sul University, Rua Galvão Bueno, 868, 13° andar, Bloco B, 01506-000 São Paulo, SP, Brazil

## Abstract

**Background:**

Body weight support (BWS) systems on treadmill have been proposed as a strategy for gait training of subjects with stroke. Considering that ground level is the most common locomotion surface and that there is little information about individuals with stroke walking with BWS on ground level, it is important to investigate the use of BWS on ground level in these individuals as a possible alternative strategy for gait training.

**Methods:**

Thirteen individuals with chronic stroke (four women and nine men; mean age 54.46 years) were videotaped walking on ground level in three experimental conditions: with no harness, with harness bearing full body weight, and with harness bearing 30% of full body weight. Measurements were recorded for mean walking speed, cadence, stride length, stride speed, durations of initial and terminal double stance, single limb support, swing period, and range of motion of ankle, knee, and hip joints; and foot, shank, thigh, and trunk segments.

**Results:**

The use of BWS system leads to changes in stride length and speed, but not in stance and swing period duration. Only the hip joint was influenced by the BWS system in the 30% BWS condition. Shank and thigh segments presented less range of motion in the 30% BWS condition than in the other conditions, and the trunk was held straighter in the 30% BWS condition than in the other conditions.

**Conclusion:**

Individuals with stroke using BWS system on ground level walked slower and with shorter stride length than with no harness. BWS also led to reduction of hip, shank, and thigh range of motion. However, this system did not change walking temporal organization and body side asymmetry of individuals with stroke. On the other hand, the BWS system enabled individuals with chronic stroke to walk safely and without physical assistance. In interventions, the physical therapist can watch and correct gait pattern in patients' performance without the need to provide physical assistance.

## Background

Mobility reestablishment is one of the main goals of a rehabilitation program for individuals with stroke [1-3]. Among the different strategies of gait training for these individuals, the use of treadmill with partial body weight support (BWS) has been a very popular one [4,5]. The theoretical background of this strategy originated from treadmill gait training in animals with a complete spinal cord injury [6,7] which established that the treadmill promotes an automatic locomotor pattern, generated by spinal neurons, named the central pattern generator [8-10].

Usually, the BWS system consists of a treadmill and a mounting frame with an apparatus in which the patient is mechanically supported by a harness while walking on a treadmill [11]. The BWS system unloads body weight symmetrically from the lower limbs as they move forward [5,12], improves balance control, and avoids falls [9].

Among the possible percentages of body weight unloading allowed by BWS systems, most studies have adopted 30% BWS because of its effectiveness on gait training [13-15]. In addition to the appropriate percentage of body weight unloading employed during gait training with BWS, it would be reasonable to evaluate the surface the patient walks on during the intervention as specifically as possible in order to facilitate skill transfer to daily life activities [10,16]. For example, the requirements for walking on treadmill differ in terms of propulsion and balance control [17] from the requirements for walking overground. In addition, the speed adopted to walk on treadmill is not self-selected as when walking overground [12,18-21].

The differences between walking on treadmill and overground have been examined in healthy adults [18,21-23] and individuals with stroke [12,19]. The different requirements of treadmill and overground walking influence gait characteristics such as joint angles, temporal-spatial parameters [18,24,25], foot contact [20], and muscle activation [12]. Similarly, these differences may also influence the ways these improvements from walking training on the treadmill are transferred to overground walking [10,21,25]. To our knowledge, only a few studies have been conducted to examine the use of BWS on ground level [15,26], and these investigations were limited to a few aspects of walking itself. Considering that ground level is the most common locomotion surface and that there is little information about individuals with stroke walking with BWS on ground level, it is important to investigate the use of BWS on ground level in these individuals as a possible alternative strategy for gait training. Therefore, the purpose of this study was to investigate individuals with chronic stroke, walking overground with BWS. More specifically, we analyzed the spatial-temporal parameters and patterns and range of motion of joint and segmental angles during ground level walking at self-selected and comfortable speeds, with and without the use of BWS, for individuals with chronic stroke. We suggest that individuals with stroke walking with BWS on ground level would show a more stable and symmetrical walking pattern.

## Methods

### Participants

Twenty-five individuals with chronic stroke from a waiting list for the university physical therapy clinic were contacted by phone and invited to take part in the study. Seventeen of these individuals agreed to be evaluated in the laboratory. After the initial evaluation, which consisted of personal data registration and physical examination (evaluation of the level of spasticity and functional gait capacity), thirteen individuals (four women and nine men), mean age, 54.46 (± 8.58) years and at intervals longer than one year since last stroke, were eligible to participate in the study. Six individuals had right-side and seven had left-side hemiparesis of either ischemic (n = 11) or hemorrhagic (n = 2) origin.

Inclusion criteria were: elapsed time since stroke longer than one year; ability to walk approximately 10 m with or without assistance; and spasticity classified under level 3 by the Modified Ashworth Scale (for more detail, see Lindquist et al. [13]). Participants were excluded if they did not present spasticity (n = 1) or did present clinical signs of heart failure (New York Heart Association), arrhythmia, or angina pectoris; orthopedic (n = 2) or other neurological diseases (n = 1) that compromised gait; or severe cognitive or communication impairments. The University ethics committee approved this study and all individuals signed an informed consent agreement.

### Task and procedures

Participants were assessed walking at a self-selected comfortable speed along a 10 m walkway in three different conditions: walking freely with or without assistance ("no harness" condition); walking with harness and full body weight bearing ("0% BWS" condition); and walking with harness and 30% of full body weight unloaded ("30% BWS" condition). Before the evaluation in each condition, all participants practiced for a few trials until they felt comfortable with the experimental conditions. Then, six trials in each condition were videotaped by four digital cameras (Panasonic, AG-DVC7P) at 60 Hz that were positioned bilaterally in order to allow simultaneous kinematic measurement of nonparetic and paretic limbs in either direction of motion (from left to right and vice-versa). In addition, one calibration trial for each experimental condition was videotaped wherein participants stood upright on the center of the walkway facing both directions for a few seconds to register the neutral position data of the joints and segments for further normalization of the joint and segmental angles.

**Figure 1:**
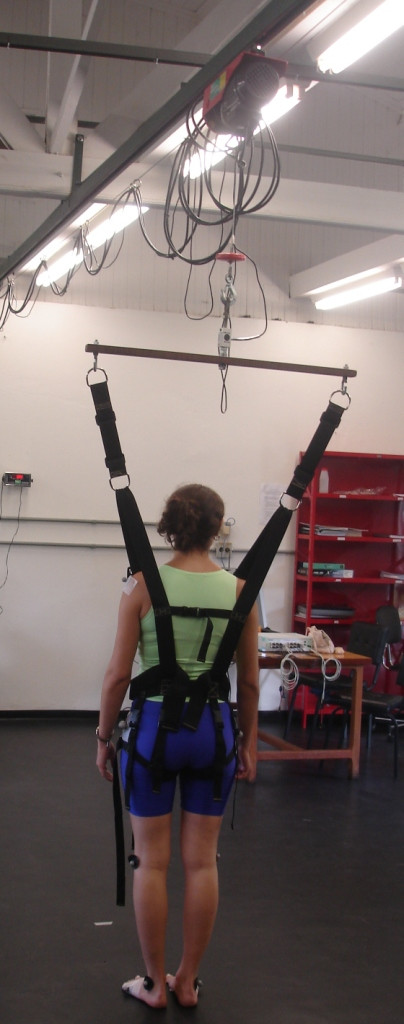
**Partial view of the body weight support system used in the study**. The rail that the electric motor slides along, the load cell, and one of the experimenters wearing the harness are shown.

During the trials using the BWS system, participants were mechanically supported in a harness with adjustable belts and padded straps for the thighs, similar to the one used by Norman et al. [17], which was attached to a horizontal bar. A steel cable from an electric motor pulled the horizontal bar upward and slid it through an upper rail as the participants walked. A load cell connected the horizontal bar to the cable and measured the amount of weight borne by the BWS system, which was shown on a digital display. In order to support the weight, participants stayed still until the motor was activated by the experimenter, who lengthened or shortened the cable to bear the desired amount of body weight. Figure 1 illustrates the BWS system used in the present study.

Passive reflective markers were placed on the nonparetic and paretic sides of the body at the following anatomical locations: head of the fifth metatarsal, lateral malleolus, lateral epicondyle of the femur, greater trochanter, and acromion, in order to define the foot, shank, thigh, and trunk segments, respectively. The digitalization and the reconstruction of all markers were performed using Ariel Performance Analysis System - APAS (Ariel Dynamics, Inc.) software, and filtering and posterior analyses were performed using Matlab software (MathWorks, Inc. - Version 6.5). Reconstruction of the real coordinates was performed using the direct linear transformation (DLT) procedure.

### Data analysis

One intermediate stride per trial by each participant, for a total of three selected trials for each condition, was analyzed. The trial selection was determined by the best visualization of the markers and walking performance in an uninterrupted trial. Through visual inspection, a stride (walking cycle) was defined by two consecutive initial contacts of the same limb to the ground along the progression line. In addition, walking events during a stride were identified for subsequent calculation of walking temporal organization (initial and terminal double stance, single limb support, and swing period [27]). This procedure was carried out for both nonparetic and paretic sides of the body

All the data were digitally filtered using a 4^th ^order and zero-lag Butterworth filter and all markers were low-pass filtered at 8 Hz. For joint and segmental angles, strides were normalized in time from 0 to 100%, with a 1% step. These cycles were referenced to the participants' neutral angles measured during the calibration trial in each condition and were then averaged to obtain the mean cycle for each participant. The same procedure was repeated to obtain the mean cycle among participants.

The following variables were examined: mean walking speed, calculated as the ratio between the distance traveled and its duration (determined by the position of the greater trochanter marker, which is closer to the center of body mass); stride length, the distance between two successive initial contacts of each foot to the ground (determined by the position of the lateral malleolus marker); stride speed, calculated as the ratio between stride length and duration; durations of total double stance and single limb support; ankle, knee, and hip joint range of motion, calculated from the difference between the maximum and minimum angles of these joints during each stride cycle; and foot, shank, thigh, and trunk segment range of motion, calculated from the difference between the maximum and minimum angles of these segments during each stride cycle. The movements of the segments were counter-clockwise (backward) and clockwise (forward) rotations around the medial-lateral axis on the sagittal plane, which denoted positive and negative values, respectively [28]. For example, a counter-clockwise rotation of the trunk means trunk extension from neutral position and a clockwise rotation means trunk flexion from neutral position.

### Statistical analysis

For all variables, data from three trials under each condition were averaged for each participant. A one-way analysis of variance (ANOVA) was conducted, using the three experimental conditions (no harness, 0% BWS, 30% BWS) as factors. Four multivariate analyses of variance (MANOVAs) were employed, using body side (nonparetic and paretic) and the three experimental conditions as factors. The dependent variables were mean walking speed for the ANOVA, cadence, stride length, and stride speed for the first MANOVA; durations of initial double stance, single limb support, terminal double stance, and swing period for the second MANOVA; ankle, knee, and hip joint range of motion for the third MANOVA; and foot, shank, thigh, and trunk segmental range of motion for the fourth MANOVA. When applicable, univariate analyses and Tukey post hoc tests were employed. An alpha level of 0.05 was adopted for all statistical tests, which were performed using SPSS software (Version 10.0).

## Results

All participants performed the requested tasks. None used assistive devices during walking performance; however, three participants needed assistance from a physical therapist that hold one of their hands, in order to support balance when walking with no harness. The results for walking spatial-temporal parameters and for joint and segmental pattern and range of motion follow.

### Temporal-spatial gait parameters

Table 1 depicts mean and standard deviation (± SD) of the walking cycle temporal-spatial parameters. Walking speed was different among conditions, F(2,24) = 5.56, p = 0.02, in which it was lower in the 30% BWS than in the no harness condition. MANOVA revealed condition significance, Wilks' Lambda = 0.54, F(6,44) = 2.65, p = 0.003, and condition and body side interaction, Wilks' Lambda = 0.37, F(6,44) = 4.68, p = 0.001. Univariate analyses indicated condition effect and condition and body side interaction for stride length, F(2,24) = 8.39, p = 0.007, F(2,24) = 12.41, p < 0.001, and stride speed, F(2,24) = 4.96, p = 0.029, F(2,24) = 16.31, p < 0.001, respectively. Stride length was shorter and stride speed was lower in the 30% BWS than in the no harness and 0% BWS conditions, and in the 0% BWS than in the no harness condition. The paretic side displayed longer stride length and faster stride speed than the nonparetic side only in the 30% BWS condition (Table 1).

**Table 1:** Temporal-spatial parameters of walking during the stride cycle.

Outcome measures	Conditions					
	
	No Harness		0% BWS		30% BWS	
	
	Nonparetic	Paretic	Nonparetic	Paretic	Nonparetic	Paretic
Walking speed (m/s)	0.41 ± 0.24^a^		0.38 ± 0.23		0.30 ± 0.14^a^	
Cadence (steps/min)	70.82 ± 20.63	70.73 ± 22.32	70.53 ± 21.02	71.48 ± 22.19	69.50 ± 14.53	72.13 ± 14.52
Stride Length (m)	0.63 ± 0.20	0.63 ± 0.20^a^	0.58 ± 0.19	0.58 ± 0.18^b^	0.48 ± 0.16	0.52 ± 0.16^a, b^
Stride Speed (m/s)	0.40 ± 0.22	0.40 ± 0.23^a^	0.36 ± 0.22	0.37 ± 0.22^b^	0.28 ± 0.12	0.32 ± 0.13^a, b^
Initial double stance (%)	26.59 ± 11.71	21.93 ± 12.52	27.11 ± 11.33	22.42 ± 11.53	27.68 ± 9.66	19.14 ± 7.92
Single limb support (%)	32.19 ± 9.34^1^	18.49 ± 7.02^1^	30.56 ± 9.19^2^	18.09 ± 7.05^2^	31.56 ± 8.30^3^	18.42 ± 5.13^3^
Terminal double stance (%)	21.82 ± 9.58	26.46 ± 12.07	23.54 ± 10.84	27.53 ± 11.73	22.23 ± 8.27	28.69 ± 9.16
Swing period (%)	19.40 ± 6.76^1^	33.12 ± 10.18^1^	18.79 ± 7.13^2^	31.96 ± 9.81^2^	18.54 ± 4.87^3^	33.75 ± 8.74^3^

Mean (± SD) values of mean walking speed, cadence, stride length, stride speed, duration of initial double stance, single limb support, terminal double stance, and swing period, during stride cycle in the three conditions (no harness, 0% of BWS, and 30% of BWS) on nonparetic and paretic body sides of individuals with chronic stroke (n = 13). Note: same letter indicates difference between conditions; same number indicates difference between body sides.

Regarding temporal measures, MANOVA only revealed significant body side effect, Wilks' Lambda = 0.14, F(4,9) = 13.71, p = 0.001. Univariate analyses indicated that the nonparetic side displayed longer single limb support, F(1,12) = 53.36, p < 0.001, and shorter swing period duration, F(1,12) = 65.88, p < 0.001, than the paretic side of the body (Table 1).

### Joint and segmental angles

**Figure 2:**
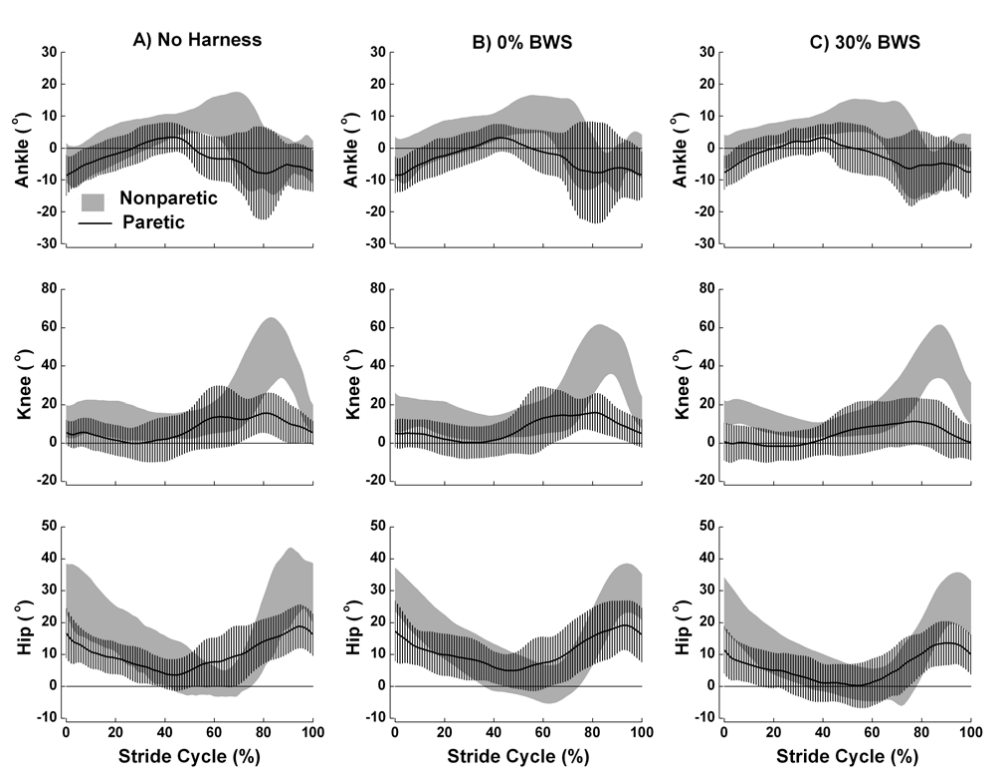
**Ankle, knee, and hip joint angles during the stride cycle**. Mean (± SD) stride cycle of ankle, knee, and hip joint angles for the individuals with chronic stroke walking with no harness (A), with 0% BWS (B), and 30% BWS (C) on nonparetic (gray area) and paretic (line) body sides. Positive values denote ankle dorsiflexion, knee and hip flexion, and negative values denote ankle plantar flexion, knee and hip extension (n = 13).

Figure 2 shows the mean (± SD) stride cycle of ankle, knee, and hip angle patterns in the three conditions (no harness, 0% BWS, and 30% BWS) for paretic and nonparetic sides of the body. Qualitatively, the joints of either side have a similar pattern amongst conditions. However, joint angles between sides presented a remarkably different pattern.

The ankle joint of the paretic side showed plantar flexion during most of the gait cycle, and little dorsiflexion during middle stance (approximately 40% of the cycle) in the three conditions (Figure 2, upper panel). On the other hand, the ankle of nonparetic side showed marked dorsiflexion later in the cycle. The knee joint (Figure 2, middle panel) showed little flexion on the paretic side considering that this joint on the nonparetic side presented a much larger flexion at swing period (approximately 85% of gait cycle) in the three conditions. Finally, the hip joint (Figure 2, bottom panel) showed a flexor pattern with little extension during the entire cycle for both sides. However, the hip on the nonparetic side showed greater flexion than the hip on the paretic side in the three conditions.

Table 2 depicts mean (± SD) joint range of motion during the walking cycle. MANOVA revealed joint range of motion had significant difference for conditions, Wilks' Lambda = 0.52, F(6,44) = 2.87, p = 0.02, body side, Wilks' Lambda = 0.09, F(3,10) = 33.73, p < 0.001, and condition and body side interaction tendency, Wilks' Lambda = 0.58, F(6,44) = 2.28, p = 0.053. The hip joint was influenced by condition, F(2,24) = 10.49, p = 0.004, with a greater range of motion in the no harness condition than in the 30% BWS condition and a greater range of motion in the 0% BWS than in the 30% BWS condition. Range of motion was greater on the nonparetic side for the ankle, F(1,12) = 21.98, p = 0.001, knee, F(1,12) = 41.91, p < 0.001, and hip, F(1,12) = 102.97, p < 0.001, than in the paretic side (Table 2).

**Table 2:** Joint and segmental range of motion during the stride cycle.

Outcome measurements	Conditions					
	
	No Harness		0% BWS		30% BWS	
	
	Nonparetic	Paretic	Nonparetic	Paretic	Nonparetic	Paretic
**Joint (degrees)**						
Ankle	26.22 ± 4.53^1^	18.23 ± 9.62^1^	25.73 ± 6.03^2^	17.90 ± 8.95^2^	25.83 ± 7.36^3^	17.40 ± 9.43^3^
Knee	51.76 ± 6.23^1^	27.25 ± 13.81^1^	49.32 ± 5.43^2^	26.91 ± 14.32^2^	47.38 ± 6.46^3^	24.74 ± 14.05^3^
Hip	34.27 ± 6.49^1^	19.72 ± 5.87^1, a^	32.73 ± 5.58^2^	18.21 ± 5.79^2, b^	29.43 ± 5.75^3^	17.72 ± 6.18^3, a, b^
**Segment (degrees)**						
Foot	58.67 ± 12.77^1^	37.90 ± 14.88^1^	54.03 ± 11.76^2^	36.45 ± 14.40^2^	51.74 ± 13.40^3^	34.20 ± 15.61^3^
Shank*	50.25 ± 9.80^1,4,5^	34.21 ± 11.30^1,6,7, a^	45.74 ± 8.77^2,4^	32.10 ± 11.23^2,6,8, a, b^	39.62 ± 7.92^3,5^	29.65 ± 11.69^3,7,8, a, b^
Thigh	31.43 ± 4.87^1^	26.52 ± 5.79^1, a^	29.75 ± 3.98^2^	24.65 ± 5.31^2, a, b^	26.51 ± 3.98^3^	21.10 ± 5.97^3, a, b^
Trunk*	8.23 ± 2.48	12.77 ± 3.11^1^	7.88 ± 2.90	11.82 ± 3.53^2^	7.91 ± 2.77	9.35 ± 3.45^1,2^

Mean (± SD) values for range of motion of ankle, knee, and hip joints, and foot, thigh, shank, and trunk segments during stride cycle in the three conditions (no harness, 0% of BWS, and 30% of BWS) on nonparetic and paretic body sides of individuals with chronic stroke (n = 13). Note: same letter indicates difference between conditions; same number indicates difference between body sides; * indicates interaction

**Figure 3:**
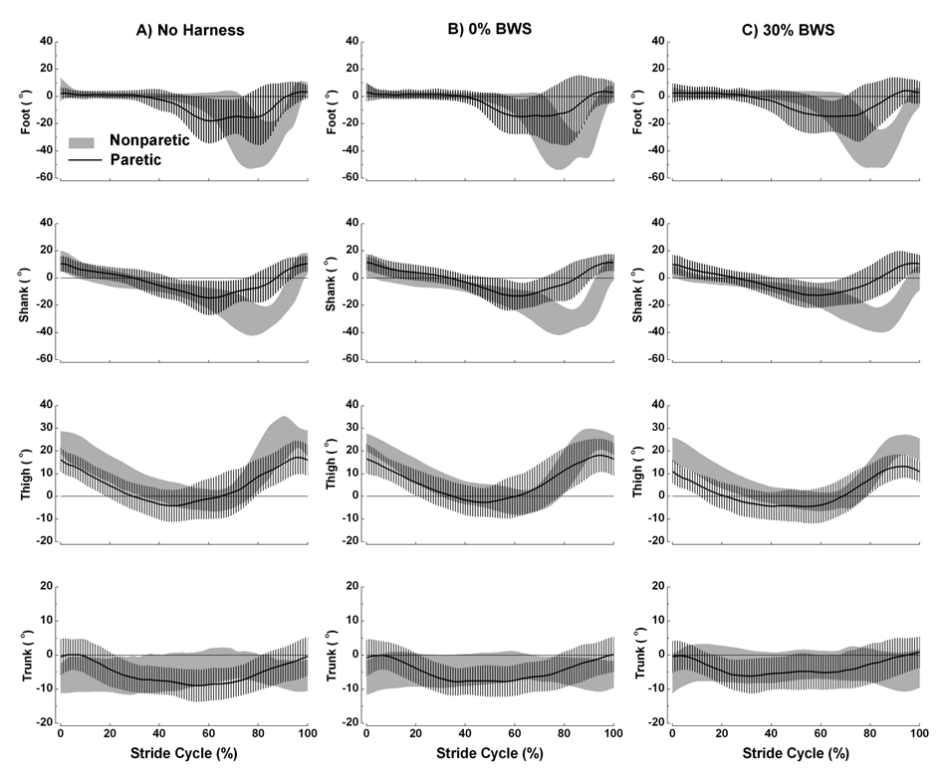
**Foot, shank, thigh, and trunk segmental angles during the stride cycle**. Mean (± SD) stride cycle of foot, shank, thigh, and trunk segmental angles for the individuals with chronic stroke walking with no harness (A), with 0% BWS (B), and 30% BWS (C) on nonparetic (gray area) and paretic (line) body sides. Positive values denote counter-clockwise (backward) rotation of segments and negative values denote clockwise (forward) rotation of segments (n = 13).

Figure 3 shows the mean (± SD) stride cycle of foot, thigh, shank, and trunk angle patterns in the three conditions for paretic and nonparetic sides of the body. Most of segmental angles displayed similar pattern in the three conditions, however, there were some different patterns between sides.

The foot remained close to neutral position during most of the stance period on both sides. The foot on the nonparetic side presented greater clockwise rotation and later than the foot on the paretic side in all conditions (Figure 3, upper panel). The same pattern was observed for shank. The thigh was the only segment that presented a similar pattern between nonparetic and paretic sides during most of the gait cycle. The thigh on the nonparetic side showed a more counter-clockwise rotation than the thigh on the paretic side (Figure 3, middle panel), except at the end of the swing period. Finally, the trunk presented an opposite orientation between nonparetic and paretic sides and was close to neutral position with 30% BWS (Figure 3, bottom panel).

Table 2 also displays mean (± SD) segmental range of motion during the walking cycle. MANOVA revealed segmental range of motion significant difference for condition, Wilks' Lambda = 0.35, F(8,42) = 3.67, p = 0.003, body side, Wilks' Lambda = 0.13, F(4,9) = 14.85, p = 0.001, and condition and body side interaction, Wilks' Lambda = 0.24, F(8,42) = 5.54, p < 0.001. Condition influenced thigh range of motion, F(2,24) = 17.08, p = 0.001, with greater range of motion in the no harness than in the 0% and 30% BWS conditions and greater range of motion in the 0% BWS than in the 30% BWS condition. Body side influenced foot, F(1,12) = 35.77, p < 0.001, and thigh, F(1,12) = 22.34, p < 0.001, range of motion with both segments showing a greater range of motion on the nonparetic than on the paretic side. Finally, condition and body side interaction was observed for the shank, F(2,24) = 20.40, p < 0.001, and trunk, F(2,24) = 8.08, p = 0.007, range of motion. Shank range of motion was decreased throughout the no harness, 0% BWS, and 30% BWS conditions on both sides, but with a greater decrease on the nonparetic than on the paretic side. Trunk range of motion was decreased throughout the no harness, 0% BWS, and 30% BWS conditions only on the paretic side and presented a smaller range of motion on the nonparetic side in the no harness and 0% BWS conditions than on the paretic side (Table 2).

## Discussion

This study investigated spatial-temporal gait parameters, and joint and segmental angles of individuals with chronic stroke walking at self-selected comfortable speed on ground level with and without BWS. The results revealed that the use of BWS system leads to changes in stride length and stride speed of individuals with chronic stroke, but not on stance and swing period duration. Regarding the joint range of motion, the hip was the only joint that was influenced by the BWS system with the paretic side presenting less hip joint range of motion during walking in the 30% BWS condition than in the no harness condition, and the nonparetic side presenting less hip joint range of motion in the 30% BWS than in the no harness and 0% BWS conditions. Finally, regarding the segmental range of motion, shank and thigh segments presented less range of motion in the 30% BWS condition than in the other conditions and less range of motion in the 0% BWS condition than in the no harness condition. The trunk on the paretic side presented less range of motion in the 30% condition than in the other conditions and difference between paretic and nonparetic sides was only observed in the 30% BWS condition. These results did not support our initial suggestion that an individual with stroke walking with BWS on ground level would present a more stable and symmetrical gait pattern.

At first glance, it seems that individuals with chronic stroke had more difficulty walking with BWS on ground level than without it. However, one of the most important issues regarding this study is that the BWS system enabled these individuals to perform the task on a surface that is used in daily life activities and none required assistance to keep their balance because the BWS system enabled them to walk by themselves safely. In interventions, the BWS provides physical support instead of the physical therapist, who can then focus attention on the patient's walking performance. For example, the physical therapist can focus on increased walking speed and its influence on spatial-temporal parameters and joint patterns [9] in the patient and correct gait pattern to favor a more symmetrical gait [10,29]. The BWS system also provided steadiness during the single limb support on the paretic side which led to a greater joint range of motion during stepping. These results are quite encouraging for gait training using BWS on ground level on a long-term basis.

Another positive aspect of walking with BWS on ground level is the better vertical alignment of the trunk throughout gait cycle (Figure 3, bottom panel). We had investigated the trunk segment from both sides of the body in the sagital plane of motion because of the posture that individuals with stroke usually adopt for walking. This segment presented different ranges of motion between nonparetic and paretic sides, which means that the individuals rotated the trunk (longitudinal axis of motion) towards the opposite side, which presented the largest range of motion. In the 30% BWS condition, the trunk was close to neutral position (i.e. erect) and did not present any difference between nonparetic and paretic sides for range of motion. Trunk positioning is a critical aspect of gait pattern, as its alignment is related to functional performance [30], and it might contribute to a decreased mechanical energy cost [31]. Therefore, BWS on ground level contributes to aligning the trunk and provides advantages during gait performance.

Contrary to previous investigation of walking with BWS on ground level [15], the participants in this study walked slower in the 30% BWS than in the no harness condition. This difference might be attributed to the different procedures adopted in each case. While Lamontagne and Fung [15] investigated individuals with acute stroke and classified them according to their walking speed as either low or high functioning individuals, we evaluated individuals with chronic stroke and did not classify them according to their preferred walking speed. Also, we did not encourage our patients to speed up along the pathway, as Lamontagne and Fung [15] did and also had evaluated their participants with stroke walking at preferred walking and maximal walking speed.

Slow walking speed in the 30% BWS condition would be due to decreased posterior muscle energy generation by the lower limb at the end of terminal double stance. This aspect has been described as fundamental to propel the limb forward to control the walking speed [32]. We had adopted 30% BWS for this study as it has been the most common percentage of body weight support used during gait training with BWS on treadmill and it was the percentage used in the previous study on ground level [15]. However, it seems that this percentage for walking with BWS on ground level might not be as appropriate as it is for walking with BWS on treadmill, because it may prevent ground reaction force generation and, consequently, the impulse to move the limb forward. In this way, future studies using BWS on ground level in individuals with chronic stroke should investigate a more appropriate percentage of body weight support for this type of surface. Further, BWS systems that can be modulated dynamically according to the gait phase have been proposed for treadmill [33] and should also be considered for ground level.

An unexpected finding was a longer stride length on the paretic side than on the nonparetic side in the 30% BWS condition. Any human walking on a straight line should present the same stride length on both sides [34], but this was not the case in the present study. One possible explanation for this finding could be that individuals with chronic stroke took advantage of the body weight support on the single limb of the nonparetic side to generate a longer and quicker step with the paretic limb.

Our results, as in the previous investigation [15], also showed that BWS itself did not change gait asymmetry between nonparetic and paretic sides among the experimental conditions, which is a prominent characteristic of hemiparetic gait [35,36]. However, it is possible that side asymmetry might decrease only after a gait training period with BWS on ground level, although this hypothesis still needs to be further investigated.

Last but not least, the 0% BWS did not influence the mean walking speed, temporal symmetry, ankle, knee, foot, and trunk ranges of motion. Although the harness was employed mainly to help with balance, it also contributed to shortening the stride length, lowering stride speed, and reducing hip, shank, and thigh range of motion when compared to the no harness condition. These reductions were lower in the 0% BWS condition than in the 30% BWS condition. Thus, the use of harness itself was already enough to change the gait pattern of individuals with stroke. This result might be due to the BWS system adopted in this study because it required the individuals to move the motor along the rail and to a lack of sufficient adaptation to this walking requirement before taking part in the study. In future studies, use of a BWS system for ground level in which the motor is moved along the rail by a specific controller rather than by the participant wearing the harness, should be considered. Actually, we are currently working on the system in order to implement such a condition.

To our knowledge, this was the first study that considered a more detailed description of walking with BWS on ground level in individuals with stroke and it presented some limitations. First, a full understanding of gait requires more analyses than just the kinematic approach, such as kinetic and electromyographyc analyses. Second, the need to move the motor through the rail by the participants creates a drag force as they walked and this can influence walking performance and pattern. Third, only the 0% and 30% of BWS were analyzed and participants might take advantage of other percentages of body weight unloading especially due to the difficulty in force production to move forward in the 30% of BWS condition. Finally, the adaptation period provided to the participants might have not been long enough and this could have masked some of the effects of BWS use. Despite all these limitations, the use of BWS system overground seems to be a useful and important strategy as a tool to provide an alternative intervention and rehabilitation program for individuals with stroke.

## Conclusion

Individuals with stroke using BWS system on ground level walked slower and with shorter stride length and slower stride speed, respectively, than with no harness. BWS also led to a reduction in hip, shank, and thigh range of motion. However, this system did not change walking temporal organization and the body side asymmetry of individuals with stroke. The differences found in this study might be attributed to the adjustments the individuals had to make to walk with an unloading condition on the lower limb, and to the brief period of adaptation to the BWS system, as the use of the harness without support of body weight (0% BWS condition) per se leads to some alterations during the task performance.

Although the use of BWS system on ground level changed some gait parameters, this system enabled individuals with chronic stroke to walk safely and without physical assistance. In interventions, the physical therapist can focus on watching and correcting the individual's gait pattern during performance instead of providing physical assistance.

## Competing interests

The authors declare that they have no competing interests.

## Authors' contributions

COS and AMFB were responsible for conception and design of the study, acquisition of data, analysis and interpretation of data, and drafting the article. CLPM was responsible to acquisition of data, analysis and interpretation of data, drafting the article. TFS and JAB were responsible for interpretation of data and revising it critically for scientific method and content. All authors read and approved the final manuscript.
